# Aortic Stenosis assessment with a 3-directional phase contrast magnetic resonance technique. Comparison to transthoracic echocardiography

**DOI:** 10.1186/1532-429X-17-S1-P381

**Published:** 2015-02-03

**Authors:** Juliana Serafim da Silveira, Matthew Smyke, Ning Jin, Rizwan Ahmad, Lua Jafari, Debbie Scandling, Jennifer A Dickerson, Subha V Raman, Orlando P Simonetti

**Affiliations:** 1 Dorothy M. Davis Heart and Lung Research Institute, The Ohio State University, Columbus, OH, USA; 2 College of Engineering, The Ohio State University, Columbus, OH, USA; 3 Siemens Healthcare, Columbus, OH, USA; 4 College of Medicine, The Ohio State University, Columbus, OH, USA; 5 Department of Internal Medicine/Division of Cardiovascular Medicine, The Ohio State University, Columbus, OH, USA; 6 Department of Radiology, The Ohio State University, Columbus, OH, USA

## Background

Transthoracic Doppler-echocardiography (TTE) is the standard clinical method for diagnosis and staging of aortic stenosis (AS). AS staging is based on measurement of aortic peak velocity, transvalvular gradient, and calculation of aortic valve area. Unidirectional through-plane phase-contrast magnetic resonance imaging (1DPC-MRI) has been widely applied in clinical imaging to quantify aortic peak velocities and flow. Nonetheless, 1DPC-MRI requires accurate positioning of imaging planes perpendicular to flow direction in order to avoid peak velocity underestimation, which can be challenging in patients with multiple or eccentric jets. Therefore PC techniques with multi-directional velocity quantification would likely improve the accuracy of velocity determination, and allow for more accurate grading of AS severity. The aim of this study is to determine whether a rapid technique that is able to capture 3 directions of velocity in a 2D image plane in a single breath-hold (3DPC-MRI) provides more accurate estimation of diagnostic parameters compared with the traditional 1DPC-MRI, using TTE as the reference standard.

## Methods

We included 13 patients diagnosed with mild to severe AS by TTE (nine men, age range: 39-85 years, median age: 65 years). The average time elapsed between TTE and CMR was 24 days. Velocity-encoded CMR included breath-hold 1DPC-MRI and 3DPC-MRI. Acquisition parameters are listed in Table 1. After manual tracing of aortic valve contours, quantitative image analysis was performed offline using custom software developed in MATLAB (Mathworks, Natick, MA). The pixel with the highest average velocity within the valve contour was used to extract aortic peak velocities and peak and mean trans-valvular gradients, for comparison with TTE. Agreement between CMR and TTE parameters were explored using intraclass correlation coefficient (ICC). Statistical analyses were performed using SPSS, version 21 (IBM).

## Results

**Figure 1:**
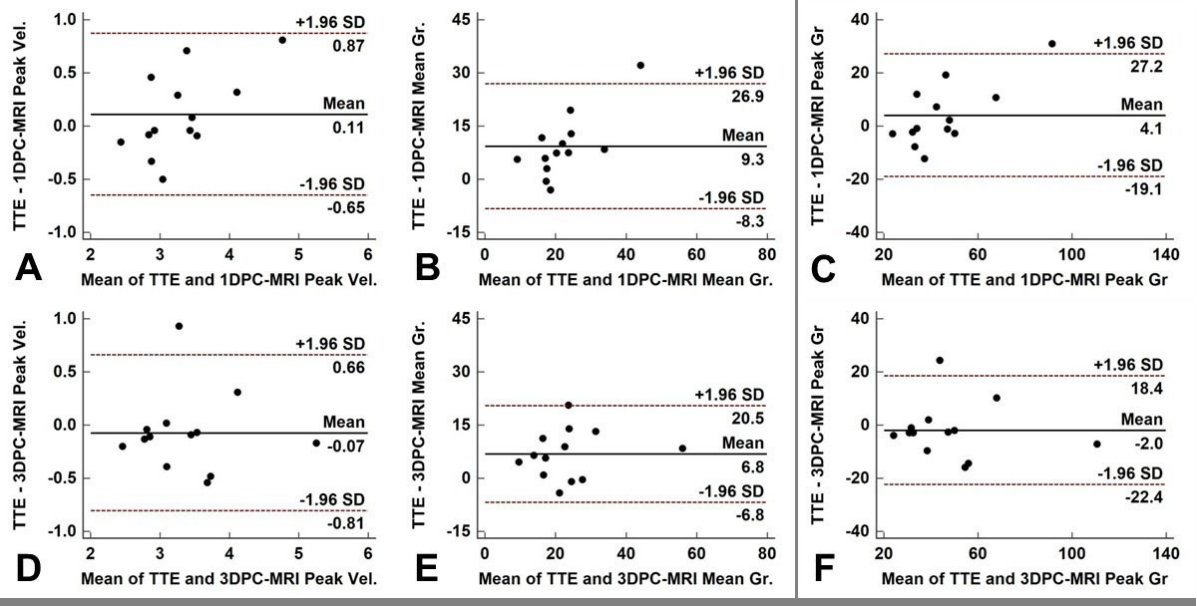
Bland-Altman plots of comparison between parameters derived from TTE versus 1DPC-MRI (A-C) and 3DPC-MRI (D-F).

3DPC-MRI peak velocities showed higher correlation with TTE (ICC 0.88, p<0.001), than 1DPC-MRI (ICC 0.82, p<0.001). 3DPC-MRI mean gradient estimation also showed better correlation with TTE results (ICC 0.72, p<0.001) than 1DPC-MRI (ICC 0.42, p=0.013). Since peak gradient estimations derive from peak velocity estimations, 3DPC-MRI peak gradients again showed better correlation with TTE (ICC 0.90, p<0.001) than 1DPC-MRI (ICC 0.80, p<0.001). Bland-Altman plots between TTE vs 1DPC-MRI (A-C), and TTE vs 3DPC-MRI parameters (D-F) are shown in Figure 1. Note less overall bias for the 3DPC-MRI technique.

## Conclusions

Initial results in a small patient cohort support the hypothesis that 3DPC-MRI provides better estimation of hemodynamic parameters in AS patients in comparison to 1DPC-MRI.

## Funding

Research grant from Siemens.

**Table 1:** Imaging parameters.

Parameter	1DPC-MRI	3DPC-MRI
TR (ms)	52.25	49.56

TE (ms)	2.06	2.75

Flip Angle	25°	15°

Bandwidth (Hz/pixel)	420	1860

Typical In-plane Resolution (mm)	2.3 x 1.8	2.3 x 1.8

Slice Thickness (mm)	6.0	6.0

Triggering	Prospective	Prospective

Acquisition time (s)	10-14	10-14

